# Fatty acid oxidation and autophagy promote endoxifen resistance and counter the effect of AKT inhibition in ER-positive breast cancer cells

**DOI:** 10.1093/jmcb/mjab018

**Published:** 2021-03-23

**Authors:** Lei Duan, Sarah Calhoun, Daeun Shim, Ricardo E Perez, Lothar A Blatter, Carl G Maki

**Affiliations:** 1 Department of Cell and Molecular Medicine, Rush University Medical Center, Chicago, IL 60612, USA; 2 Department of Molecular Biophysics and Physiology, Rush University Medical Center, Chicago, IL 60612, USA

**Keywords:** endoxifen, fatty acid oxidation, autophagy, AMPK, AKT

## Abstract

Tamoxifen (TAM) is the first-line endocrine therapy for estrogen receptor-positive (ER+) breast cancer (BC). However, acquired resistance occurs in ∼50% cases. Meanwhile, although the PI3K/AKT/mTOR pathway is a viable target for treatment of endocrine therapy-refractory patients, complex signaling feedback loops exist, which can counter the effectiveness of inhibitors of this pathway. Here, we analyzed signaling pathways and metabolism in ER+ MCF7 BC cell line and their TAM-resistant derivatives that are co-resistant to endoxifen using immunoblotting, quantitative polymerase chain reaction, and the Agilent Seahorse XF Analyzer. We found that activation of AKT and the energy-sensing kinase AMPK was increased in TAM and endoxifen-resistant cells. Furthermore, ERR**α**/PGC-1**β** and their target genes MCAD and CPT-1 were increased and regulated by AMPK, which coincided with increased fatty acid oxidation (FAO) and autophagy in TAM-resistant cells. Inhibition of AKT feedback-activates AMPK and ERR**α**/PGC-1**β**-MCAD/CPT-1 with a consequent increase in FAO and autophagy that counters the therapeutic effect of endoxifen and AKT inhibitors. Therefore, our results indicate increased activation of AKT and AMPK with metabolic reprogramming and increased autophagy in TAM-resistant cells. Simultaneous inhibition of AKT and FAO/autophagy is necessary to fully sensitize resistant cells to endoxifen.

## Introduction

Estrogen receptor-positive (ER+) breast cancer (BC) accounts for ∼70% of total BC patients. The mainstay therapy for ER+ BC patients is endocrine therapy including tamoxifen (TAM) and aromatase inhibitors that suppress ER activation to block BC cell proliferation and induce apoptosis. TAM is a selective estrogen receptor modulator that has antagonistic and agonistic effects on ERα and is the first-line therapy choice for premenopausal patients. TAM is clinically effective and has greatly reduced recurrence and prolonged the lives of millions of patients over the last three decades. However, resistance to TAM develops in as many as 50% of women taking this endocrine therapy, which prevents successful treatment of the patients (Early Breast Cancer Trialists’ Collaborative Group, 1998).

Multiple pathways and molecular mechanisms have been identified, which promote TAM resistance. Foremost among these is the PI3K/AKT/mTORC1 pathway (Kirkegaard et al., 2005; Miller et al., 2011; Bostner et al., 2013; Mills et al., 2018; Vasan et al., 2019). Activation of the PI3K/AKT/mTORC1 pathway promotes proliferation, survival, and cell growth. Heightened activation of this pathway is common in TAM resistance, and inhibitors of the pathway can sensitize BC cells to TAM. Notably, overexpression of a constitutive active AKT can promote TAM resistance in ER+ MCF7 cells (Campbell et al., 2001). The PI3K inhibitor alpelisib and the mTOR inhibitor everolimus are currently Food and Drug Administration-approved for treatment of endocrine-refractory ER+ BC. Autophagy is another mechanism for TAM resistance (Cook et al., 2011; Duan et al., 2011; Duan et al., 2014; Liu et al., 2019). Autophagy is a self-eating process in which cells degrade damaged proteins and internal organelles in autophagolysosomes and shuttle the degradation products into metabolic pathways needed for survival. TAM treatment can activate autophagy, and autophagy inhibitors have been reported to increase TAM sensitivity (Qadir et al., 2008). Alterations in energy metabolism have also been linked to TAM resistance. Glycolysis and mitochondrial respiration can be monitored by measuring the extracellular acidification rate (ECAR) and oxygen consumption rate (OCR) in cells, respectively. Fiorillo et al. (2018) reported that TAM-resistant cells had heightened OCR and increased ATP production compared to TAM-sensitive counterparts. Others reported the oncoprotein MUC1 can promote TAM resistance in part by increasing expression of proteins that promote cholesterol synthesis (Poirot et al., 2012). These latter findings suggested a possible role for cholesterol and/or lipid metabolism in TAM resistance. Yet another mechanism linked with TAM resistance is the expression of estrogen-related receptor alpha (ERRα) (Thewes et al., 2015). ERRα has sequence homology with ERα and like ERα can bind to DNA and function as a transcription factor. However, ERRα has no known ligand and is thus considered an orphan receptor (Audet-Walsh and Giguére, 2015; Huss et al., 2015). ERRα promotes transcription in conjunction with coactivactor proteins peroxisome proliferator activated receptor gamma coactivator 1alpha (PGC-1α) and PGC-1β (Schreiber et al., 2004). Increased ERRα expression was linked to poor outcome in TAM-treated BC patients, suggesting that it may contribute to TAM resistance (Thewes et al., 2015).

Crosstalk between the pathways that promote TAM resistance could potentially impact cancer cell responses to TAM and reduce TAM effectiveness. Central to this crosstalk is AKT and the energy-sensing kinase AMPK. Activated AMPK can influence metabolism by increasing glycolysis, while at the same time inhibiting mTORC1 to block cell growth and increase autophagy (Hardie, 2011a, b; Herzig and Shaw, 2018). Recent studies suggested that AMPK can mediate AKT activation in response to different stresses such as hypoxia, glucose deprivation, and H_2_O_2_ treatment (Leclerc et al., 2010; Han et al., 2018; Lieberthal et al., 2019). Activated AKT can also increase glycolysis by, for example, increasing translocation of glucose transporters to the cell membrane (Cong et al., 1997). However, unlike AMPK, activated AKT promotes mTORC1 activity leading to increased growth and reduced autophagy (Noguchi et al., 2014; Butler et al., 2017). AKT has also been reported to inhibit AMPK in mouse cardiac myocytes and tumor cells by phosphorylating Ser487 (Kovacic et al., 2003; Hawley et al., 2014). It is unclear at present whether or how the crosstalk between AKT and AMPK influences autophagy, metabolism, and TAM sensitivity in BC. The current study was undertaken to address this question.

## Results

### TAM-resistant cells are cross-resistant to endoxifen

TAM is a prodrug metabolized in the liver to generate active metabolites such as 4-hydroxytamoxifen (4OHTAM) and endoxifen that can inhibit cell proliferation at nanomolar concentrations and induce apoptosis at micromolar concentrations (Mandlekar and Kong, 2001). Phase I study with endoxifen in BC patients showed that pharmacological concentrations of endoxifen in plasma reach the 5 µM range without observable toxicity to the patients (Goetz et al., 2017). We previously generated TAM-resistant derivative cells (TRC) from ER+ MCF7 cells by treating MCF7 cells for prolonged periods with 4OHTAM (Duan et al., 2011, 2014). To test whether these cells are also resistant to endoxifen, we treated MCF7 cells and TRC with increasing doses of enodoxifen and then analyzed for proliferation/viability by MTT assay and apoptosis by determining the percentage of cells with sub-G1 DNA content. MCF7 cells showed a dose-dependent decrease in MTT absorbance in response to endoxifen (Supplementary Figure S1A) and an increase in the sub-G1 cell population at high doses (5 and 10 µM) of endoxifen (Supplementary Figure S1B), suggesting that endoxifen can inhibit cell proliferation and induce apoptosis. However, endoxifen failed to significantly decrease MTT absorbance (Supplementary Figure S1A) or induce apoptosis (Supplementary Figure S1B) in TRC, indicating that TRC are resistant to endoxifen. Notably, the MTT results are normalized to the untreated control in both MCF7 cells and TRC to reflect the response differences in these cells.

### AKT, AMPK, and ERRα contribute to endoxifen resistance

**Figure 1:**
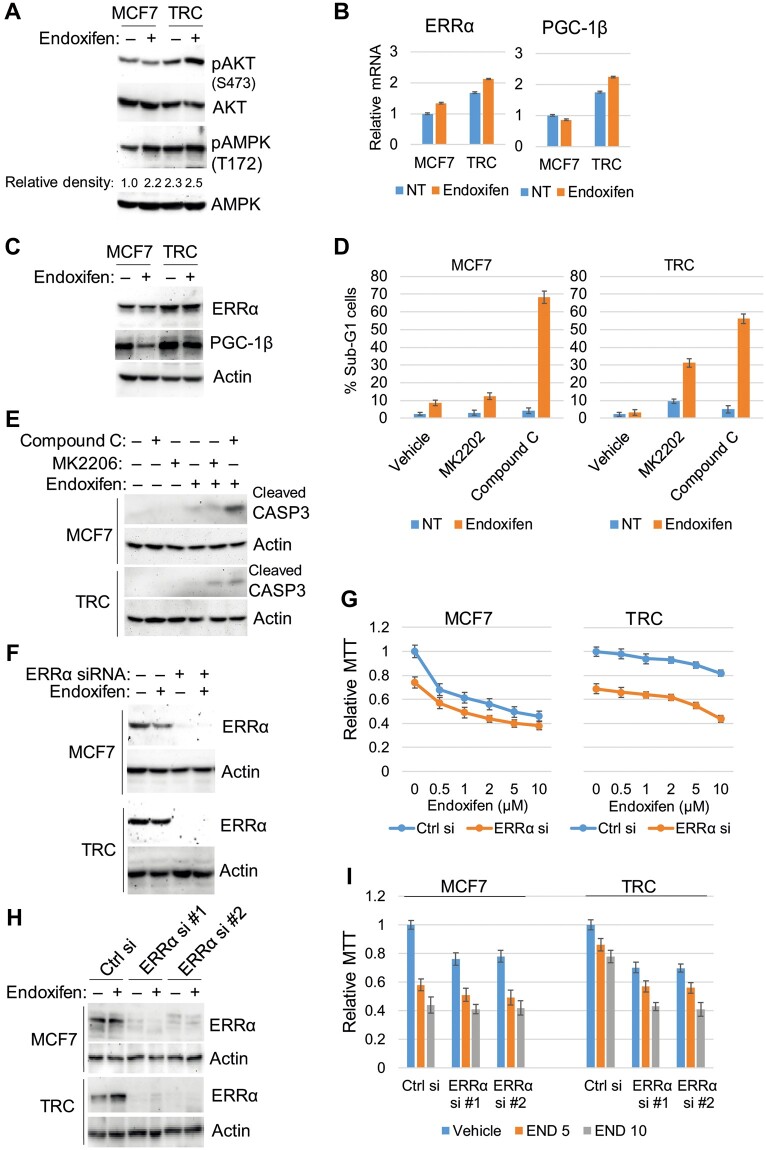
AKT/AMPK and ERRα regulate cell survival and endoxifen resistance. (**A**‒**C**) MCF7 cells and TRC were treated with vehicle or endoxifen (5 µM) for 24 h. (**A** and **C**) Lysates were immunoblotted for the indicated proteins. Relative density of pAMPK normalized to control is indicated below the blot. (**B**) mRNA expression was analyzed with quantitative polymerase chain reaction (qPCR) for the indicated genes. There are significant differences (*P *<* *0.05) in ERRα and PGC-1β mRNA levels between MCF7 cells and TRC basally (NT, no treatment) and in response to endoxifen. (**D** and **E**) MCF7 cells and TRC were treated with vehicle or endoxifen (5 µM) with or without MK2206 (5 µM) or compound C (10 µM) for 72 h. (**D**) The cells were analyzed for sub-G1 cells. Average (triplicate) % sub-G1 (apoptotic) cells is presented with SD indicated. (**E**) Lysates were immunoblotted for cleaved caspase-3 and actin. (**F** and **G**) The cells were transfected with control siRNA or ERRα siRNA (pool) and then treated with vehicle or endoxifen (5 µM) for 24 h for western blotting analysis (**F**) or treated with vehicle or the indicated doses of endoxifen for 72 h for MTT analysis (**G**). Relative average (eight replicates) MTT absorbance is presented with SD indicated. (**H** and **I**) The cells were transfected with control siRNA or two individual pairs of ERRα siRNA (#1 and #2) and then treated with vehicle or endoxifen (5 µM) for 24 h for western blotting analysis (**H**) or treated with vehicle or endoxifen (5 or 10 µM) for 72 h for MTT analysis (**I**). There are significant differences (*P *<* *0.05) between control siRNA and ERRα siRNA (#1 and #2)-transfected TRC treated with endoxifen.

We next compared levels of phosphorylated (activated) AKT and AMPK in endoxifen-treated MCF7 cells and TRC. We also compared the expression of ERRα and its transcriptional coactivator PGC-1β in these cells. As shown in Figure 1A, levels of activated AKT (S473-phosphorylated) and activated AMPK (T172-phosphorylated) were higher in TRC than in MCF7 cells both basally and in response to endoxifen. ERRα and PGC1β levels (mRNA and protein) were also higher in TRC than in MCF7 cells basally and in response to endoxifen (Figure 1B and C). We used gene knockdown or pharmacologic inhibitor approaches to examine whether AKT, AMPK, and ERRα contribute to TAM resistance. As shown in Figure 1D and E, treatment with the allosteric AKT inhibitor MK2206 had little effect in MCF7 cells but sensitized TRC to endoxifen, evidenced by a large increase in sub-G1 cells and cleavage of caspase-3. In contrast, the AMPK inhibitor compound C caused a pronounced sensitization to endoxifen in both MCF7 cells and TRC. Moreover, ERRα knockdown with a pooled ERRα siRNA or two individual ERRα siRNAs reduced proliferation/viability in both MCF7 cells and TRC as determined by MTT assay and appeared to cause an additive reduction in proliferation/viability when combined with endoxifen (Figure 1F‒I). The results indicate that both AKT and AMPK contribute to endoxifen resistance in TRC, and AMPK also contributes to endoxifen resistance in MCF7 cells. ERRα appears important for basal proliferation and viability in MCF7 cells and TRC and has an additive inhibitory effect when combined with endoxifen.

### TRC display metabolic reprogramming that includes heightened glycolysis and increased fatty acid oxidation

**Figure 2:**
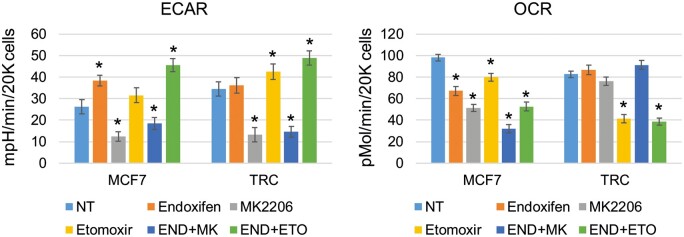
TRC have increased FAO and glycolysis. MCF7 cells and TRC were seeded on 96-well plates and pretreated with endoxifen (END, 5 µM), MK2206 (MK, 5 µM), etomoxir (ETO, 50 µM), END plus MK, or END plus ETO for 24 h and analyzed with the Agilent Seahorse XF Analyzer for ECAR and OCR. Average (six replicates) ECAR (mpH/min/20000 cells) and OCR (pMol/min/20000 cells) are presented with SD indicated. * indicates a significant difference (*P *<* *0.05) between the NT and treated conditions.

While AMPK can increase glycolysis, it can also increase fatty acid oxidation (FAO), a process whereby fatty acids are transported into and metabolized in the mitochondria to yield ATP. FAO can be monitored in cells by measuring mitochondrial OCR in the medium that does not contain glucose or glutamine and in the presence or absence of specific FAO inhibitors such as etomoxir. We used the Agilent Seahorse XF Analyzer to measure ECAR and OCR in MCF7 cells and TRC treated with endoxifen alone or with the AKT inhibitor MK2206 or the FAO inhibitor etomoxir. The results shown in Figure 2 revealed several differences between MCF7 cells and TRC. First, basal glycolysis (ECAR) was higher in TRC, consistent with higher levels of activated AKT and AMPK that can promote glycolysis in these cells. Second, endoxifen reduced OCR and caused a corresponding increase in ECAR in MCF7 cells, while having no effect in TRC. Third, inhibition of AKT by MK2206 alone or in combination with endoxifen decreased ECAR and OCR in MCF7 cells, while in TRC, AKT inhibition reduced ECAR but did not decrease OCR when given alone or in combination with endoxifen. This indicates that glycolysis is AKT-dependent in TRC, while mitochondrial respiration and oxygen consumption are independent of AKT (and glucose). Fourth, etomoxir decreased OCR to a far greater extent in TRC than in MCF7 cells. This result indicates that the TAM and endoxifen-resistant TRC have increased FAO compared with MCF7 cells, evidenced by a higher level of etomoxir-sensitive oxygen consumption. Notably, ECAR was increased in etomoxir-treated TRC, suggesting inhibition of FAO increases AKT-dependent glycolysis. Altogether, these results indicate a metabolic reprograming in TRC that includes increased glycolysis and increased FAO.

**Figure 3:**
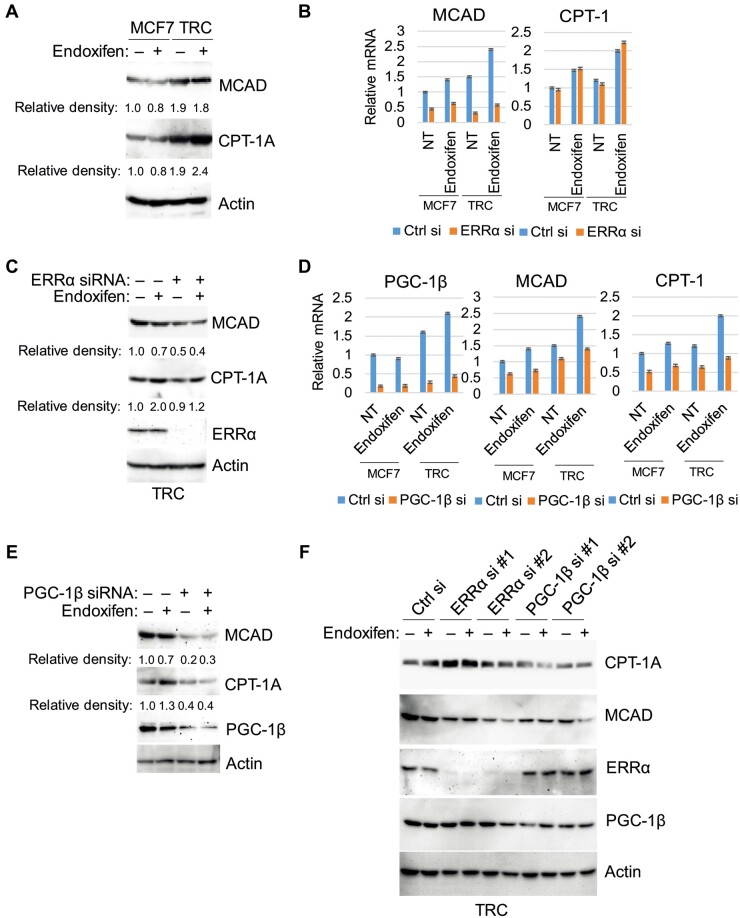
TRC express higher levels of MCAD and CPT-1A regulated by AMPK and ERRα/PGC-1β. (**A**) The cells were treated with vehicle or endoxifen (5 µM) for 24 h. Lysates were immunoblotted for the indicated proteins. (**B**‒**E**) The cells were transfected with control siRNA, ERRα siRNA (pool, **B** and **C**), or PGC-1β siRNA (pool, **D** and **E**) and then treated with vehicle or endoxifen (5 µM) for 24 h. (**B** and **D**) mRNA expression was analyzed for the indicated genes. There are significant differences (*P *<* *0.05) in MCAD and CPT-1A mRNA levels between endoxifen-treated MCF7 cells and TRC (**B**), in MCAD but not CPT-1A mRNA levels between control siRNA and ERRα siRNA-transfected MCF7 cells and TRC (**B**), and in MCAD and CPT-1A mRNA levels between control siRNA and PGC-1β siRNA-transfected MCF7 cells and TRC treated with endoxifen (**D**). (**C** and **E**) Lysates were immunoblotted for the indicated proteins. Relative density of the proteins normalized to loading control is indicated below the blots. (**F**) The cells were transfected with control siRNA, individual pairs of ERRα siRNA (#1 and #2), or individual pairs of PGC-1β siRNA (#1 and #2) and then treated with vehicle or endoxifen (5 µM) for 24 h. Lysates were immunoblotted for the indicated proteins.

**Figure 4:**
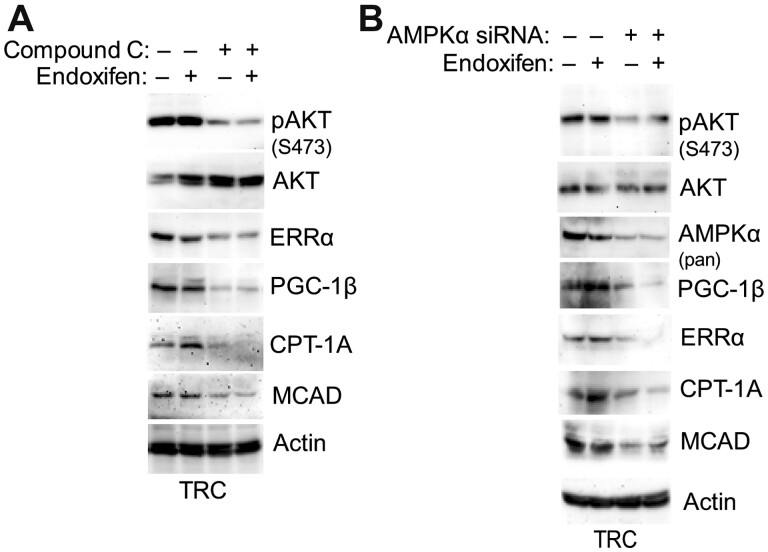
Inhibition/depletion of AMPK decrease the protein expression of ERRα, PGC-1β, MCAD, and CPT-1A. (**A**) The cells were treated with vehicle or endoxifen without or with compound C (10 µM) for 24 h. (**B**) The cells were transfected with control siRNA or AMPKα1/α2 siRNA and then treated with vehicle or endoxifen (5 µM) for 24 h. Lysates were immunoblotted for the indicated proteins.

ERRα and its coactivator PGC-1 can transcriptionally activate a range of mitochondrial genesis and lipid metabolism genes including *CPT-1* and *MCAD* whose protein products increase FAO by promoting either the transport of fatty acids into the mitochondria (CPT-1) or their breakdown/oxidation in the mitochondria (MCAD) (Sladek et al., 1997; Sadana et al., 2007). AMPK has been reported to regulate and promote expression of ERRα and PGC-1 (Hu et al., 2011; Audet-Walsh et al., 2016). Based on these, we hypothesized that increased FAO in TRC may result from AMPK-mediated expression of ERRα and PGC-1β and subsequent expression of MCAD/CPT-1. To test this, we asked whether MCAD and CPT-1 expression levels are increased in TRC and whether it is dependent on ERRα and/or PGC-1β. Immunoblotting showed both MCAD and CPT-1A protein levels are higher basally and in response to endoxifen in TRC than in MCF7 cells (Figure 3A). Next, MCF7 cells and TRC were transfected with either control siRNA or siRNA against ERRα or PGC-1β, and the ability of endoxifen to induce MCAD and CPT-1A expression was assessed. As shown in Figure 3B, both MCAD and CPT-1A mRNA levels were increased by endoxifen treatment in control MCF7 cells and TRC. However, knockdown of ERRα with a pooled ERRα siRNA blocked the induction of MCAD mRNA expression by endoxifen but had no effect on CPT-1A. In contrast, PGC-1β knockdown with a pooled siRNA blocked the increase in MCAD and CPT-1A mRNA and protein levels in response to endoxifen (Figure 3D and E). Furthermore, knockdown with two individual siRNAs showed that knockdown of ERRα reduced MCAD but not CPT-1A protein levels, while knockdown of PGC-1β reduced both CPT-1A and MCAD protein levels (Figure 3F). These results suggest that ERRα is important for MCAD expression, whereas PGC-1β is important for both MCAD and CPT-1A expression basally and in endoxifen-treated MCF7 cells and TRC. Last, we determined whether AMPK is important for ERRα, PGC1β, MCAD, and CPT-1A expression in endoxifen-treated TRC. For this, we used compound C to inhibit AMPK or siRNA against AMPK. As shown in Figure 3A and B, both inhibition (compound C) and knockdown of AMPK reduced ERRα, PGC-1β, MCAD, and CPT-1A expression in untreated and endoxifen-treated cells. Taken together, the results suggest that AMPK promotes expression of ERRα and PGC-1β, which then promote MCAD and CPT-1A expression. Notably, inhibition/knockdown of AMPKα decreased AKT phosphorylation (Figure 4A and B). Since MCAD and CPT-1A promote FAO and are expressed at higher levels in TRC compared to MCF7 cells, we propose that increased FAO in TRC in part result from the increased ERRα/PGC-1β and subsequently increased CPT-1A/MCAD expression.

**Figure 5:**
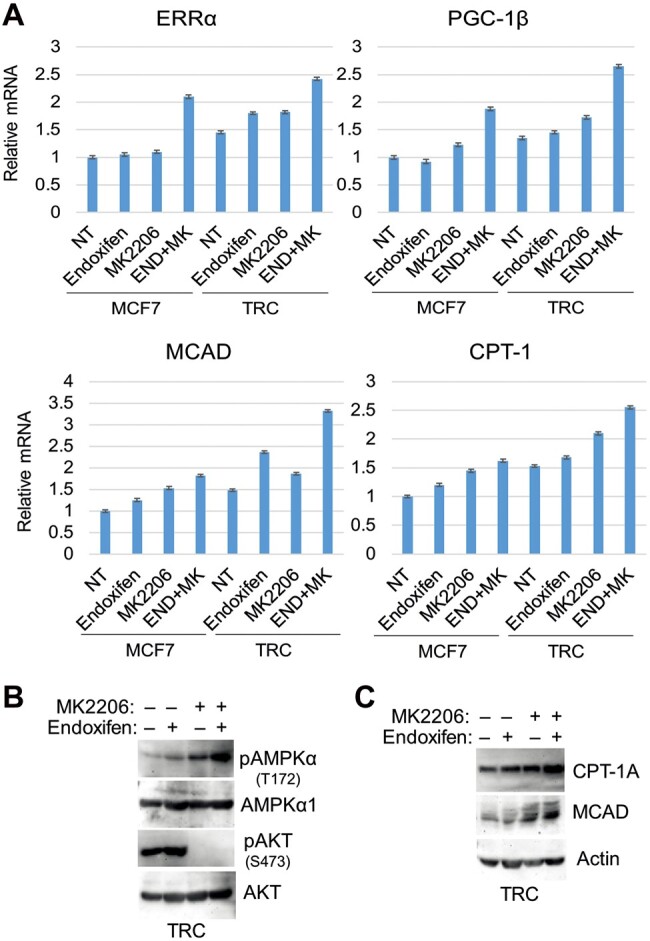
Inhibition of AKT feedback-increases the expression of MCAD and CPT-1. The cells were treated with vehicle or endoxifen (5 µM) with or without MK2206 (5 µM) for 24 h. (**A**) mRNA expression was analyzed for the indicated genes. (**B** and **C**) Lysates were immunoblotted for the indicated proteins.

**Figure 6:**
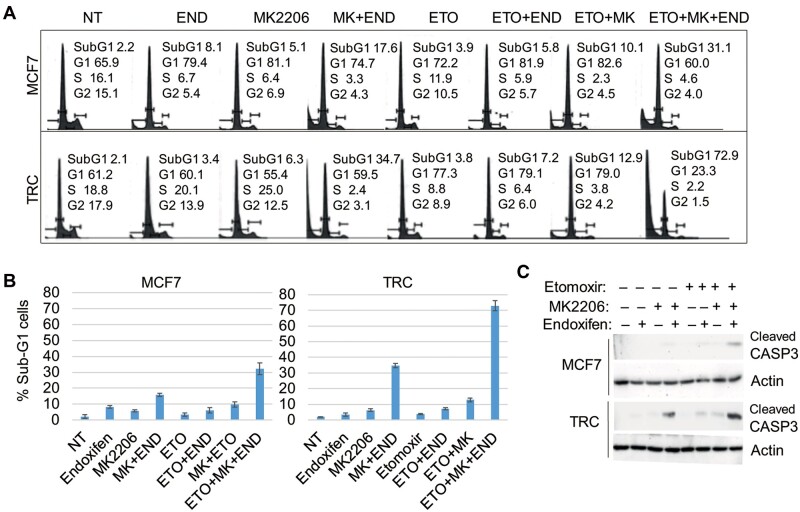
Combined inhibition of AKT and FAO overcomes endoxifen resistance. MCF7 cells and TRC were treated with vehicle, END (5 µM), MK (5 µM), ETO (10 µM), or the indicated combinations. (**A** and **B**) Cells treated for 72 h were analyzed with flow cytometry for cell cycles. (**A**) Cell cycle profiles were presented with population distribution in different phases indicated. (**B**) Average (triplicate) % sub-G1 (apoptotic) cells is presented with SD indicated. There are significant differences (*P *<* *0.01) between treatments with triple drug combination and double drug combinations in both MCF7 cells and TRC and between MCF7 cells and TRC treated with END+MK or with triple drug combination. (**C**) Cells treated for 24 h were immunoblotted for cleaved caspase-3 and actin.

Previous studies showed that AKT can inhibit AMPK by direct phosphorylation of AMPK at Ser485 (Ning et al., 2011). AKT can also downregulate and inhibit expression of PGC-1β (Southgate et al., 2005; Li et al., 2007). Based on this, we speculated that AKT may crosstalk with AMPK to regulate ERRα, PGC-1β, MCAD, and CPT-1A expression and subsequently FAO in MCF7 cells and/or TRC. To test this, we monitored mRNA levels of ERRα, PGC-1β, MCAD, and CPT-1A in MCF7 cells and TRC treated with endoxifen and/or the AKT inhibitor MK2206. As shown in Figure 5A, the mRNA levels were increased slightly by MK2206 and endoxifen alone and increased to a higher level by combined treatment. Immunoblotting showed that MK2206 blocked activation (S473 phosphorylation) of AKT but increased the activating phosphorylation (T172) of AMPK when given alone or with endoxifen (Figure 5B). CPT-1A and MCAD protein levels were also increased by MK2206 treatment either alone or with endoxifen (Figure 5C). The results suggest a possible negative feedback loop between AKT and AMPK-ERRα/PGC-1β and the regulated MCAD and CPT-1. Inhibition of AKT may suppress glycolysis (Figure 2) while simultaneously activating FAO. To determine whether co-inhibiting AKT and FAO would cause synergistic killing when combined with endoxifen in MCF7 cells and TRC, we measured cell cycle distribution and the percentage of cells with sub-G1 DNA content. As shown in Figure 6A and B, untreated MCF7 cells and TRC have similar cell cycle profiles. Endoxifen induced more MCF7 cells accumulating in G1 phase compared with TRC, consistent with reduced response of TRC to TAM (Supplementary Figure S1A). Etomoxir alone caused more TRC accumulating in G1 phase compared with MCF7 cells (Figure 6A), which is consistent with increased FAO in TRC. Etomoxir had no killing effect in MCF7 cells and TRC when given alone or in combination with endoxifen. However, combining etomoxir with endoxifen and MK2206 caused much greater killing of both MCF7 cells and TRC than endoxifen plus MK2206 alone, and this effect was significantly greater in TRC than in MCF7 cells (Figure 6A and B). Consistent with the increase in the percentage of sub-G1 cells, the triple drug combination also led to increased cleavage of caspase-3 (Figure 6C). This result suggests that FAO contributes to endoxifen resistance under conditions where AKT is inhibited. Thus, co-treatment with AKT and FAO inhibitors can effectively sensitize resistant cells to endoxifen.

### AMPK promotes autophagy, while inhibition of AKT feedback-activates autophagy

**Figure 7:**
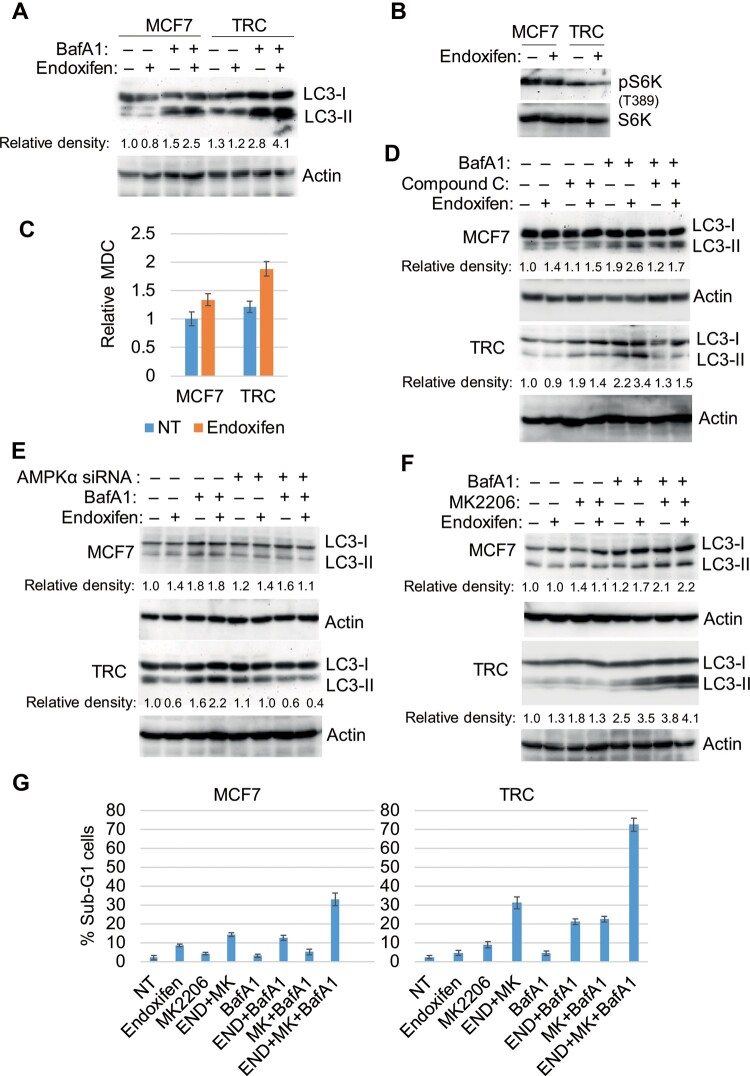
AMPK promotes autophagy that counters the effect of AKT inhibition. (**A**‒**C**) The cells were treated with vehicle or endoxifen (5 µM) with or without BafA1 (10 nM) for 24 h. Lysates were immunoblotted for the indicated proteins (**A** and **B**) and analyzed for MDC fluorescence (**C**). (**D**‒**F**) The cells were treated with vehicle or endoxifen (5 µM) with or without BafA1 for 24 h, in the presence or absence of compound C (10 µM, **D**), control siRNA or AMPKα1/α2 siRNA (**E**), or MK2206 (5 µM, **F**). Lysates were immunoblotted for the indicated proteins. Relative density of LC3-II protein normalized to loading control is indicated below the blots. (**G**) MCF7 cells and TRC were treated with vehicle, END (5 µM), MK (5 µM), BafA1 (10 nM), or the indicated combinations for 72 h and then analyzed for cell cycle. Average (triplicate) % sub-G1 (apoptotic) cells is presented with SD indicated. There are significant differences (*P *<* *0.01) between treatments with triple drug combination and double drug combinations in both MCF7 cells and TRC and between MCF7 cells and TRC treated with END+MK or with drug triple combination.

We previously reported that TRC have increased autophagy that promotes survival in response to TAM (Duan et al., 2011, 2014). Because AMPK promotes autophagy by inhibiting mTORC1, we hypothesized that increased autophagy in TRC may be regulated by AMPK and contribute to endoxifen resistance. To determine the effect of endoxifen on autophagy in MCF7 cells and TRC, autophagy flux and monodansylcadaverine (MDC) sequestration were measured. LC3-II is an autophagy protein that is degraded in autophagolysosomes. MDC fluorescence is an indicator of mature autophagolysosomes and a common measure of autophagy. Bafilomycin A1 (BafA1) blocks autophagic degradation, including degradation of LC3-II. Thus, the extent to which LC3-II accumulates in BafA1-treated cells indicates the rate at which autophagic degradation is occurring or autophagy ‘flux’. LC3-II accumulated in BafA1-treated MCF7 cells and TRC, indicating that autophagic degradation was occurring in these cells (Figure 7A). LC3-II accumulated to a greater extent in TRC than in MCF7 cells treated with BafA1 alone (Figure 7A), suggesting higher basal autophagy flux in TRC. Moreover, LC3-II accumulated to greater extent in cells co-treated with enodoxifen plus BafA1 compared to BafA1 alone, and this was more evident in TRC than in MCF7 cells (Figure 7A and D‒F). As shown in Figure 7C, endoxifen also increased MDC fluorescence more significantly in TRC than in MCF7 cells. Notably, phospho-S6K is decreased in TRC compared with that in MCF7 cells (Figure 7B), which is consistent with increased AMPK activation in TRC (Figure 1A).

We hypothesized that increased AMPK activation in TRC may be responsible for the increased autophagy flux that would promote resistance to endoxifen. To test this, we first monitored autophagy flux in TRC, where AMPK was either depleted by siRNA or inhibited by compound C. As shown in Figure 7D and E, LC3-II again accumulated to a greater extent in MCF7 cells and TRC treated with endoxifen plus BafA1 compared to BafA1 alone, confirming that endoxifen increases autophagy flux in these cells. This effect was blocked by both compound C (Figure 7D) and knockdown of AMPKα (Figure 7E) and was also more pronounced in TRC compared to MCF7 cells. These findings indicate that AMPK is required for endoxifen to increase autophagy flux, especially in TRC. Since AKT inhibition increases AMPK activation in endoxifen-treated cells (Figure 5B), we tested whether AKT inhibition also increases autophagy flux. As shown in Figure 7F, LC3-II accumulated to a greater extent in MCF7 cells and TRC treated with MK2206 plus BafA1 compared to BafA1 alone, indicating that AKT inhibition increases autophagy flux in these cells. The results support a model in which inhibition of AKT leads to activation of AMPK and a consequent increase in autophagy flux. We then examined whether increased autophagy flux counters the effect of AKT inhibition on cell responses to endoxifen. For this, MCF7 cells and TRC were treated with endoxifen in the absence or presence of MK2206 or BafA1 in different combinations. Sub-G1 analysis was used for indication of cell death. As shown in Figure 7G, MK2206 or BafA1 alone caused partial sensitization of both MCF7 cells and TRC to endoxifen. However, the combination of MK2206 plus BafA1 caused a far greater increase in cell death than either agent alone. The combination treatment-induced killing is significantly higher in TRC compared to MCF7 cells. These findings suggest that the feedback-activation of autophagy that occurs when AKT is inhibited protects cells from killing by endoxifen. Co-treatment with AKT and autophagy inhibitors can effectively sensitize resistant cells to endoxifen.

## Discussion

In response to stresses, cancer cells can activate different signaling pathways in an attempt to maintain proliferation and/or survival. Activation of these pathways can limit the effectiveness of anti-cancer therapeutic agents. The PI3K‒AKT pathway, autophagy, and alterations in metabolism can all contribute to TAM resistance in BC, and PI3K‒AKT pathway inhibitors have been approved for treatment of endocrine therapy-refractory BC patients. In this work, MCF7-derivative TRC that are co-resistant to TAM and endoxifen (the active metabolite of TAM) displayed heightened activation of AKT, heightened activation of the energy-sensing kinase AMPK, increased autophagy, and altered metabolism including increased FAO. Inhibition of AMPK reduced AKT activation, reduced autophagy, reduced expression of factors that promote FAO, and sensitized resistant cells to endoxifen. Inhibition of AKT also partially sensitized cells to endoxifen, but increased AMPK activation and autophagy, and also increased expression of the factors that promote FAO. Combined inhibition of AKT, FAO, and/or autophagy were required to fully sensitize cells to endoxifen. Together, our studies identify a novel crosstalk among AKT, AMPK, autophagy, and fatty acid metabolism that regulates BC cell responses to endoxifen. The results suggest that combination approaches to inhibit these pathways simultaneously should be considered and have implications for current clinical use of PI3K‒AKT inhibitors.

AMPK plays an important role coordinating cell growth and metabolism in response to energy stresses. Thus, AMPK is activated in response to energy stresses that reduce intracellular ATP levels, such as glucose deprivation. Activated AMPK responds to these stresses by shutting down cell growth and altering metabolism to increase ATP production. For example, by inhibiting mTORC1, activated AMPK blocks mTORC1-dependent protein translation and cell growth while simultaneously derepressing autophagy (mTORC1 normally represses autophagy) (Shackelford and Shaw, 2009). Meanwhile, activated AMPK stimulates glucose uptake, glycolysis, and the breakdown (oxidation) of fatty acids, while blocking new lipid synthesis (Hardie and Pan, 2002; Hardie et al., 2012). We found that the TAM and endoxifen-resistant cells had heightened activating phosphorylation (T172) of AMPK both basally and in response to endoxifen. Knockdown of AMPK subunits or treatment with an AMPK inhibitor caused a robust sensitization of the cells to endoxifen. Our results suggest that such sensitization to endoxifen results from a combination of reduced autophagy, reduced AKT activation, and reduced FAO. For example, AMPK inhibition reduced autophagy flux, and direct inhibition of autophagy (by BafA1 treatment) caused partial sensitization of cells to endoxifen. These results indicate that AMPK-dependent autophagy contributes to endoxifen resistance. We also found that AMPK inhibition decreases levels of activated AKT and that direct inhibition of AKT (by MK2206 treatment) causes a partial sensitization of cells to endoxifen, supporting the idea that AMPK-mediated AKT activation contributes to endoxifen resistance. Notably, recent studies from Han et al. (2018) also found that AKT is activated downstream of AMPK and suggested that AMPK activates AKT by regulating AKT K63 ubiquitination via SKP2. Contrary to K48-lined ubiquitination that leads to proteosomal degradation of proteins, ubiquitination of K63 usually promotes activation of signaling proteins. Moreover, the ER-related protein and transcription factor ERRα has been implicated in TAM resistance (Thewes et al., 2015). ERRα, along with its cofactor PGC-1, can promote expression of genes such as *CPT-1* and *MCAD* whose protein products are essential for FAO (Sladek et al., 1997; Sadana et al., 2007). In our study, expression of CPT-1 and MCAD are ERRα and PGC-1β-dependent, and inhibition of AMPK reduces ERRα and PGC-1β expression and subsequently blocks CPT-1 and MCAD expression. Inhibition of FAO (by etomoxir treatment) sensitized cells to endoxifen (at least under conditions where AKT was inhibited). These results support the idea that AMPK-dependent FAO can contribute to endoxifen resistance. We speculate that targeting AMPK may be a way to overcome TAM resistance if these results are further validated in patients. AMPK is a complex molecule with tumor-suppressive function, while it can also drive cancer (Jeon and Hay, 2012; Zadra et al., 2015; Vara-Ciruelos et al., 2019). The diabetes medicine metformin can activate AMPK and thus is proposed to be used as a cancer prevention drug. The results that AMPK-promoted FAO and autophagy contribute to TAM resistance may caution the use of metformin in patients treated with TAM. It would be interesting to take epidemiology studies in patients treated with metformin for diabetes and with TAM for BC to see whether metformin hinders TAM effectiveness.

AKT is activated downstream of PI3K and can promote survival at least in part by phosphorylating and inhibiting the activity of various proapoptotic factors (Franke et al., 2003). AKT also promotes glucose uptake by stimulating the translocation of glucose transporters to the plasma membrane (Cartee and Wojtaszewski, 2007) and can activate mTORC1 by regulating the TSC1/TSC2 complex (Dibble and Cantley, 2015). AKT is hyper-activated in cancers with activating mutations in PI3K and contributes to cancer aggressiveness and survival. The PI3K inhibitor Alpelisib blocks AKT and was recently approved for treatment of endocrine therapy-refractory BC patients with activating PI3K mutations. In our work, we found that AKT inhibition activates AMPK, evidenced by increased phosphorylation of AMPK at T172. This is consistent with previous reports showing that activated AKT normally inhibits AMPK (Kovacic et al., 2003; Hawley et al., 2014). AKT inhibition also increased autophagy and increased expression of CPT-1 and MCAD that promote FAO. The autophagy inhibitor BafA1, when combined with the AKT inhibitor MK2206, caused a robust sensitization of the cells to endoxifen. This indicates that autophagy activated upon AKT inhibition contributes to endoxifen resistance. Furthermore, the FAO inhibitor etomoxir had minimal effect on endoxifen sensitivity when given alone, but caused robust sensitization to endoxifen when combined with the AKT inhibitor MK2206, indicating that FAO contributes to endoxifen sensitivity under conditions where AKT is inhibited.

**Figure 8:**
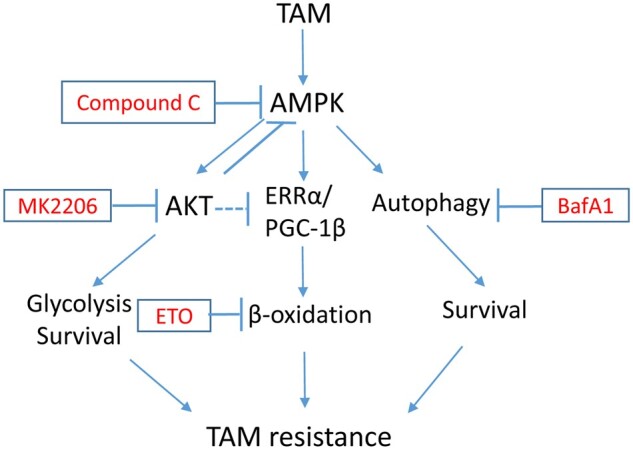
The proposed model. TAM treatment leads to activation of AMPK. Cells with chronic treatment of TAM also have elevated activation of AKT due to activation of receptor tyrosine kinases and PI3K. AMPK activation promotes AKT activation, while inhibition of AKT feedback-activates AMPK. AKT inhibition and AMPK activation promote ERRα and PGC-1β expression. AMPK additionally inhibits acetyl-CoA carboxylase. These lead to increased FAO. AMPK also promotes autophagy by direct phosphorylation of ULK1. Thus, inhibition of AKT can feedback-activate FAO and autophagy that promote cell survival and TAM resistance. Coinhibition of AKT and FAO or autophagy is necessary to overcome TAM resistance.

Our results demonstrate a novel crosstalk between AKT and AMPK that influences autophagy and metabolism (FAO) and that, in turn, dictates the cellular response to endoxifen (Figure 8). AMPK promotes AKT activation as well as autophagy and FAO that contribute to endoxifen resistance. In contrast, AKT normally inhibits AMPK, while simultaneously inhibiting apoptosis and promoting survival. Inhibition of AKT reduces apoptosis but simultaneously stimulates AMPK leading to increased autophagy and increased FAO. These findings have potential implications for guiding the use of PI3K‒AKT inhibitors against endocrine-refractory tumors, i.e. PI3K‒AKT inhibitors may inadvertently activate pro-survival AMPK, autophagy, and FAO in tumor cells, leading to reduced therapeutic benefit of these inhibitors.

## Materials and methods

### Cells and reagents

MCF7 cells were obtained from ATCC. MCF7 cells and TRC were grown in Dulbecco's modified Eagle's medium (DMEM) with 10% fetal bovine serum, penicillin (100 U/ml), and streptomycin (100 µg/ml). Cells were plated 24 h before treatment with endoxifen (Selleck Chemicals) at the indicated concentrations. 2-D-glucose (2-DG), MDC, and bafilomycin A1 were obtained from Sigma Chemical Co. MK2206 and etomoxir were obtained from Selleck Chemicals. Oligomycin, glucose, and 2-DG were prepared following manufacturer’s instructions that were supplied in the XF Glycolysis Test Kit (Seahorse Bioscience).

### Immunoblotting

Whole-cell extracts were prepared by scraping cells in lysis buffer (150 mM NaCl, 5 mM EDTA, 0.5% NP40, 50 mM Tris, pH 7.5), resolved by sodium dodecyl sulfate polyacrylamide gel electrophoresis (SDS‒PAGE) and transferred to polyvinylidene difluoride membranes (Thermo Fisher Scientific). Antibodies to p-AMPK (T172), pan AMPK, p-S6K (T389), S6K, p-AKT (S473), pan AKT, and ERRα were from Cell Signaling Technology; LC3B antibodies were from Abcam. PGC-1β antibody was from Novus Biologicals; actin antibody was from Santa Cruz. Primary antibodies were detected with goat anti-mouse or goat anti-rabbit secondary antibodies conjugated to horseradish peroxidase (Life Technologies), using Clarity chemiluminescence (BIO-RAD).

### Flow cytometry

For cell cycle analysis, cells were harvested and fixed in 25% ethanol overnight. The cells were then stained with propidium iodide (25 µg/ml, Calbiochem). Flow cytometry analysis was performed on a Gallios™ Flow Cytometer (Beckman Coulter), analyzed with FlowJo 10 (Treestar Inc.). For each sample, 10000 events were collected.

### siRNA-mediated transient knockdown

Pooled ERRα siRNA and PGC-1β siRNA (On-target plus smart pool) and control siRNA (On-target plus siControl nontargeting pool) were purchased from Dharmacon. Individual ERRα siRNAs (PDSIRNA2D: 000010 (#1), 000040 (#2)) and individual PGC-1β siRNAs (PDSIRNA2D: 000070 (#1), 000100 (#2)) were obtained from Millipore Sigma. siRNAs were transfected according to the manufacturer’s guidelines using DharmaFECT I reagent.

### RNA isolation and real-time qPCR analysis

Total RNA was prepared using Total RNA Mini Kit (IBI Scientific); the first cDNA strand was synthesized using High Capacity cDNA Reverse Transcription Kit (Applied Biosystems). Manufacturers’ protocols were followed in each case. The PCR primers for ERRα, PGC-1β, MCAD, CPT-1A, and β-actin are listed in Supplementary Table S1. SYBR Green PCR Kit (Applied Biosystems) was used according to the manufacturer’s instructions. AB7300 system was used following the conditions as follows: activation at 95°C, 2 min; 40 cycles of denaturation at 95°C, 15 sec; annealing/extension at 60°C, 60 sec; followed by melt analysis ramping from 60°C to 95°C. Relative gene expression was determined by the ΔΔ*C_t_* method using β-actin to normalize.

### Assay for glycolysis and oxygen consumption

Glycolysis and oxygen consumption were analyzed with the Agilent Seahorse XF Analyzer. Cells were seeded in culture media at 20000 cells/well of XF96 cell plate (Seahorse Bioscience) 48 h before the assay. On the day of the assay, cells were incubated in DMEM (without serum, glucose, glutamine, or bicarbonate) for 2 h before the assay in a non-CO_2_ incubator at 37°C. For endoxifen, MK2206, and etomoxir experiments, the cells were treated with the drugs 1 day before the experiment and continuously during the experiment. Injections of glucose (10 mM final concentration), oligomycin (5 µM final concentration), and 2-DG (0.1 M final concentration) were diluted in DMEM and loaded onto ports A, B, and C, respectively. The machine was calibrated and the assay was performed using glycolytic stress test assay protocol as suggested by the manufacturer (Seahorse Bioscience). The assay was run in one plate with 6–12 replicates and repeated at least three times. The rate of glycolysis is reported as ECAR (mpH/min) after the addition of glucose. The rate of oxygen consumption is reported as OCR (pMol/min).

### Quantitative analysis of autophagosomes and autolysosomes

For analysis of antophagosomes/autolysosomes, MDC sequestration was conducted as previously described (Duan et al., 2014).

### Statistical analysis

One-way analysis of variance and Student’s *t*-test were used to determine the statistical significance of differences among experimental groups. Student’s *t*-test was used to determine the statistical significance between control and experimental groups.

## Supplementary material

Supplementary material is available at *Journal of Molecular Cell Biology* online.

## Funding

This work was supported in part by a grant from the National Cancer Institute (R01CA200232-05) and a DoD breast cancer grant (11895064) to C.G.M. and by grants from National Heart, Lung, and Blood Institute (HL-057832, HL-132871, and HL-134781) to L.A.B.

**Conflict of interest:** none declared. 

**Author contributions:** L.D. and C.G.M. conceived the study. L.D. designed the experiments. L.D., S.C., D.S., and R.E.P. performed the experiments. L.A.B. and L.D. designed and performed the metabolism experiments using the Agilent XF Seahorse Analyzer. L.D. and C.G.M. wrote the manuscript.

## Supplementary Material

mjab018_Supplementary_Data
