# Corrigendum to ‘Fatty acid oxidation and autophagy promote endoxifen resistance and counter the effect of AKT inhibition in ER-positive breast cancer cells’

**DOI:** 10.1093/jmcb/mjab065

**Published:** 2022-01-28

**Authors:** Lei Duan, Sarah Calhoun, Daeun Shim, Ricardo E Perez, Lothar A Blatter, Carl G Maki

**Affiliations:** Department of Cell & Molecular Medicine, Rush University Medical Center, Chicago, IL 60612, USA; Department of Cell & Molecular Medicine, Rush University Medical Center, Chicago, IL 60612, USA; Department of Cell & Molecular Medicine, Rush University Medical Center, Chicago, IL 60612, USA; Department of Cell & Molecular Medicine, Rush University Medical Center, Chicago, IL 60612, USA; Department of Molecular Biophysics and Physiology, Rush University Medical Center, Chicago, IL 60612, USA; Department of Cell & Molecular Medicine, Rush University Medical Center, Chicago, IL 60612, USA

*Journal of Molecular Cell Biology* (2021), 13(6), 433‒444, https://doi.org/10.1093/jmcb/mjab018

In this article (p.443, the **Funding** section), the DoD grant support number listed in the original publication was incorrect. The grants that supported this work were National Cancer Institute grant (R01CA200232-05) and Department of Defense Grant (W81XWH-16-1-0025) both to C.G.M. The corrected text should be as follows:

## Funding

This work was supported in part by a grant from the National Cancer Institute (R01CA200232-05) and a Department of Defense (DoD) Grant (W81XWH-16-1-0025) both to C.G.M. and by grants from National Heart, Lung, and Blood Institute (HL-057832, HL-132871, and HL-134781) to L.A.B.

