# Supplementary material for: Fatty acid oxidation and autophagy promote endoxifen resistance and counter the effect of AKT inhibition in ER-positive breast cancer cells
Source: J Mol Cell Biol. 2021 Mar 23;13(6):433–44. doi: 10.1093/jmcb/mjab018 (PMC8436705; doi:10.1093/jmcb/mjab018)
Supplement: mjab018_Supplementary_Data [file mjab018_supplementary_data.pdf]

Supplementary material

Figure S1

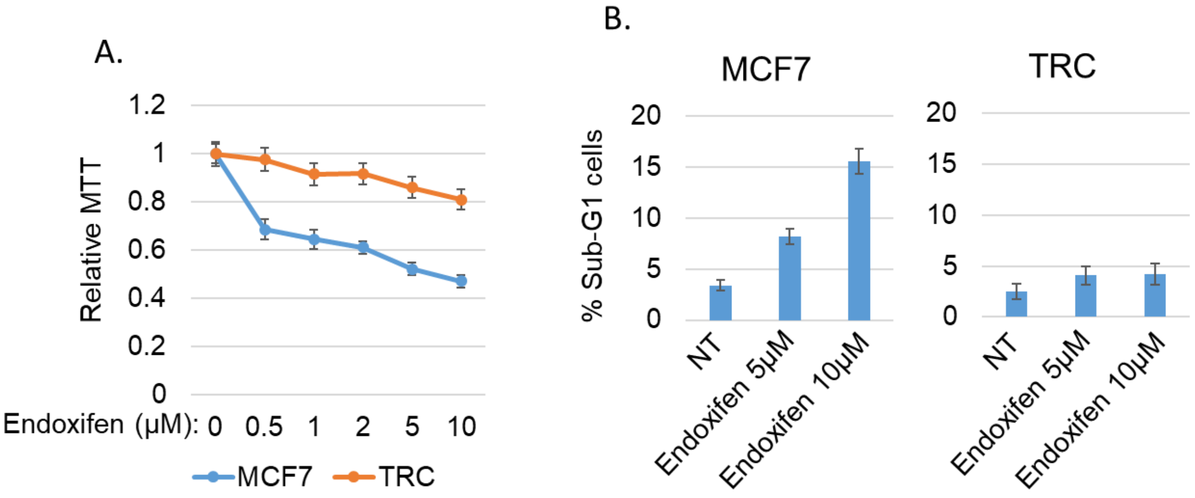

Supplementary Figure S1 TAM-resistant cells (TRCs) are resistant to endoxifen. (**A**) MCF7 cells and TRCs on 96-well plates were treated with the indicated doses of endoxifen for three days and then analyzed with MTT assay. Relative average MTT absorbance with SD (8 replicates) is presented. There is significant difference between MCF7 and TRC groups (P<0.01). (**B**) The cells were treated with endoxifen for three days and then analyzed for cell cycle with flow cytometry. Average % sub-G1 cells with SD (triplicate) is presented. There are significantly differences between vehicle and endoxifen-treated cells (P<0.05 for 5 μM and P<0.01 for 10 μM).

Table S1. PCR primer sequences

	**Primer sequences**	**Gene symbol**
Forward	ACAGGGGTTCAGACTGCTATT	*ACADM*
Reverse	TCCTCCGTTGGTTATCCACAT	*ACADM*
Forward	AGGGTTCCTCGGAGACAGAG	*ESRRA*
Reverse	TCACAGGATGCCACACCATAG	*ESRRA*
Forward	GATGCCAGCGACTTTGACTC	*PPARGC1B*
Reverse	ACCCACGTCATCTTCAGGGA	*PPARGC1B*
Forward	TCCAGTTGGCTTATCGTGGTG	*CPT-1A*
Reverse	TCCAGAGTCCGATTGATTTTTGC	*CPT-1A*
Forward	CATGTACGTTGCTATCCAGGC	*ACTB*
Reverse	CTCCTTAATGTCACGCACGAT	*ACTB*
